# Gene Expression Meta-Analysis Identifies VDAC1 as a Predictor of Poor Outcome in Early Stage Non-Small Cell Lung Cancer

**DOI:** 10.1371/journal.pone.0014635

**Published:** 2011-01-31

**Authors:** Claire Grills, Puthen V. Jithesh, Jaine Blayney, Shu-Dong Zhang, Dean A. Fennell

**Affiliations:** 1 Centre for Biomedical Informatics, Queen's University Belfast, Belfast, Northern Ireland, United Kingdom; 2 Centre for Cancer Research and Cell Biology, Queen's University Belfast, Belfast, Northern Ireland, United Kingdom; University of Giessen Lung Center, Germany

## Abstract

**Background:**

The bioenergetic status of non-small cell lung cancer correlates with tumour aggressiveness. The voltage dependent anion channel type 1 (VDAC1) is a component of the mitochondrial permeability transition pore, regulates mitochondrial ATP/ADP exchange suggesting that its over-expression could be associated with energy dependent processes including increased proliferation and invasiveness. To test this hypothesis, we conducted an in vivo gene-expression meta-analysis of surgically resected non-small cell lung cancer (NSCLC) using 602 individual expression profiles, to examine the impact of VDAC1 on survival.

**Methodology/Principal Findings:**

High VDAC1 expression was associated with shorter overall survival with hazard ratio (HR) = 0.6639 (95% confidence interval (CI) 0.4528 to 0.9721), p = 0.035352 corresponding to 52 versus 101 months. VDAC1 predicted shorter time to recurrence and was shown to be an independent prognostic factor compared with histology, gender, age, nodal stage and tumour stage in a Cox multivariate analysis. Supervised analysis of all the datasets identified a 6-gene signature comprising HNRNPC, HSPA4, HSPA9, UBE2D2, CSNK1A1 and G3BP1 with overlapping functions involving regulation of protein turnover, RAS-RAF-MEK pathway and transcription. VDAC1 predicted survival in breast cancer and myeloma and an unsupervised analysis revealed enrichment of the VDAC1 signature in specific subsets.

**Conclusions:**

In summary, gene expression analysis identifies VDAC1 gene expression as a predictor of poor outcome in NSCLC and other cancers and is associated with dysregulation of a conserved set of biological pathways, which may be causally associated with aggressive tumour behaviour.

## Introduction

Non-small cell lung cancer (NSCLC) is a common, largely incurable cancer. In the early disease setting, surgical resection is the standard of care and the benefit of adjuvant chemotherapy is limited to 5–15% improvement in survival in stage II but not stage I disease [1]. However relapse is frequent in stage I cancer with 37% of patients dying within five years, therefore there is a need to identify reliable biomarkers of poor outcome in order to target individuals for whom adjuvant or novel targeted therapy may improve survival. Alteration in tumour metabolism is a hallmark of cancer and high levels of hexokinase-dependent glucose trapping in NSCLC evidenced by standardized uptake values on positron emission tomography, is associated with shorter survival [2]. This is seen in both early and advanced NSCLC [3], [4].

The Voltage Dependent Anion Channel 1 (VDAC1) is an outer mitochondrial membrane protein involved in the regulation of ATP/ADP exchange and respiratory control [5]. VDAC1 interacts with proapoptotic BCL-2 family proteins [6], [7]. However, its role in regulating mitochondrial outer membrane depolarization has been controversial [8], [9], [10]. Yet, emerging data has identified a potential role for this protein in the regulation of cell survival and for this reason we found it worthy of further study. The permeability transition pore, which comprises VDAC1, is directly regulated by the BH3-only proapoptotic BCL-2 family member BAD [10] and regulates cell survival. In contrast, the type II isoform (VDAC2) has been identified as an antiapoptotic, negative regulator of BAK [9], [11], [12]. The adenine-nucleotide translocator (ANT) which is located in the inner mitochondrial is also considered to be a major component of the permeability transition pore [13] and along with VDAC it is believed to control mitochondrial remodeling processes either directly or indirectly [14]. Recently, the bacterial protein FilA [15] has been shown to regulate a complex of VDAC1 with hexokinase II [16] which inhibits apoptosis. A growing body of data suggests that hexokinase-VDAC interaction is anti-apoptotic [17], and disruption of this interaction could be a strategy for inducing cell death [18], [19]. VDAC-hexokinase complex has been reported to be regulated by glycogen synthase kinase 3 beta [19] and AKT [20].

Cancer cells with low VDAC1 induced by RNA interference exhibit a defect in cell growth in vivo [21]. Conversely, high levels of VDAC1 expression levels may confer tumour cell selection advantage by facilitating energy dependent processes such as proliferation and invasiveness [22]. Although hexokinase II expression has been linked to worse prognosis [23], to date there have been no studies exploring the potential prognostic impact of VDAC1 in patients with cancer. It was therefore hypothesized that VDAC1 expression could impact survival following NSCLC resection. Here, we show using gene expression meta-analysis that VDAC1 overexpression is a strong, independent, poor prognostic factor in early stage non-small cell lung cancer based on combined analysis of multiple gene expression datasets. VDAC1 overexpression is consistently associated with overexpression of 6 additional genes involved in survival signalling, protein ubiquitination and ATP binding. This association is maintained across multiple independent datasets and across different cancers suggesting a potential role for these biological processes in regulating tumour behaviour and therefore clinical outcome in NSCLC.

## Results

### VDAC1 overexpression predicts shorter survival in NSCLC

**Figure 1:**
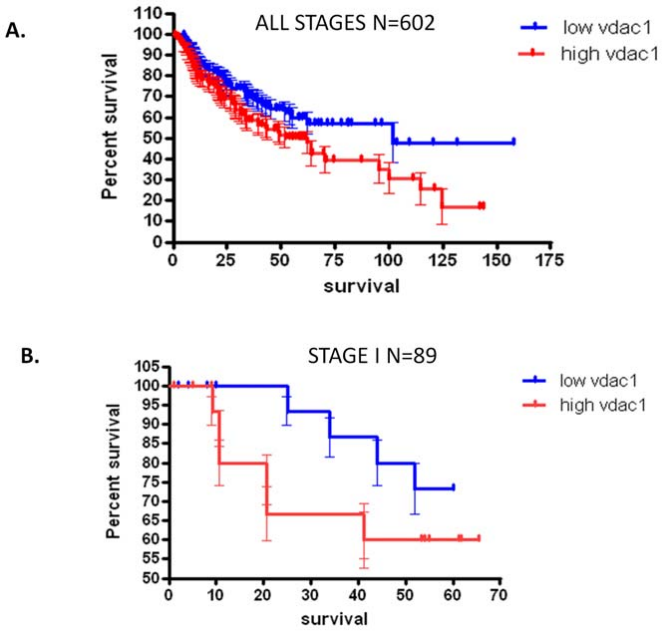
Survival curves for all data and for Stage I patients based on VDAC1 expression. Figure 1A: Displays the Kaplan Meier plot depicting patient survival dependent on high and low VDAC1 expression. The distinct difference in survival is visibly evident with the median survival of high VDAC1 expressing patients being only half that of low expressing patients. The curves are significantly different with p = 0.0353 by the logrank test. Figure 1B: The survival plot for stage I patients. Again the difference in high and low expressing samples is clear with low VDAC1 patients having better survival rates. The logrank test proves the curves are significantly different with p = 0.0455. Stage I samples follow the same pattern as all the combined data from all stages.

In total, 8 independent NSCLC datasets were curated from Gene Expression Omnibus (GEO), with a total of 602 microarrays. In all datasets high expression of VDAC1 (upper tertile) was associated with significantly shorter survival compared to that of the lower tertile for VDAC1 expression, p = 0.0353, HR = 0.6639 (0.4528 to 0.9721) (Figure 1A). The average overall survival was 101.6 months in patients with the lowest VDAC1 tertile and 52 months in patients with highest VDAC1 tertile.

### VDAC1 expression is an independent prognostic factor

To further explore the impact of VDAC1 on survival, we carried out correlation and Cox regression analysis as well as multivariate tests using PASW Statistics v.18 (SPSS: An IBM company). High VDAC1 expression was shown to be an independent prognostic variable for shorter survival p<0.001 level. Stage (p = 0.013) and pT, (primary tumour), (p = 0.022) also proved to be independent variables correlated with survival, while multivariate analysis showed that the combination of stage and histology significantly influences patient survival (p = 0.027). The results from the Cox regression analysis can be seen in Table 1, where VDAC1 has the most significant influence on survival followed by pT and stage.

**Table 1:** Cox Regression analysis based on all patient data and VDAC1 expression.

	Regression Coefficient	Standard Error (SE)	p-value	Hazard Ratio
**VDAC1**	0.239	0.78	0.002	1.27 (1.09–1.48)
**Tumour Type**	0.044	0.137	0.75	1.045 (0.80–1.37)
**Sex**	−0.078	0.238	0.745	0.925 (0.58–1.48)
**Age**	−0.02	0.011	0.834	0.998 (0.98–1.02)
**Stage**	0.515	0.249	0.038	1.67 (1.03–2.73)
**pT**	1.623	0.695	0.019	5.07 (1.30–19.78)
**pN**	1.977	1.058	0.062	7.22 (0.91–57.45)

The influence VDAC1 and stage have on survival is evident from this figure with high VDAC1 expression inducing poor survival. VDAC1 is the most significant predictor of survival time with stage and pT also displaying predictive power. The age of the patient, their gender or whether the tumour was squamous or adenocarcinoma didn't have a large effect on overall survival time.

### VDAC1 overexpression predicts shorter time to recurrence following NSCLC resection

The influence of VDAC1 expression on time to relapse following surgical resection was of interest as this clinical variable is not confounded by therapy after relapse. It is therefore a more accurate measure of prognostic significance. So we analysed only the recurrence data and univariate analysis showed VDAC1 to significantly predict time to recurrence (p<0.001). Information on histological subtype, gender and age was available for all samples and they were found to be not significant with p values of 0.35, 0.82 and 0.73 respectively. A summary of this analysis can be found in Table 2. Similarly multivariate analysis ranked these variables in the same order, with VDAC1 being the only independent factor (p<0.001).

**Table 2:** Univariate Analysis of Time to Recurrence Data.

	Type III Sum of Squares	F	Sig.
VDAC1	5103.97	14.55	0.000
Tumour Type	308.37	0.88	0.35
Sex	18.17	0.52	0.82
Age	41.47	0.118	0.73

When dealing with time to recurrence VDAC1 expression was again shown to be the most significant variable at predicting time to recurrence. We found no significant relationship between recurrence and tumour type, gender or age.

### VDAC1 overexpression in Stage I samples leads to poor survival

Information on stage was not available with all the datasets but we analysed what we had access to. We had 89 Stage I samples and the Kaplan-Meier plot for these patients is shown in Figure 1B; p = 0.0455, with an associated hazard ratio of 0.52 (0.1492–0.7653). We found the correlation between VDAC1 expression and survival to be very strong with p<0.001. Cox regression analysis in stage 1 NSCLC, where adjuvant chemotherapy has not been shown to improve patient outcome [24], [25], [26], revealed no variables to be statistically significant but, similarly to the analysis of all the data, VDAC1 was the most influential predictor of survival (p = 0.241), and was ranked above histology (p = 0.344), age (p = 0.465), pT (p = 0.970), pN, (nodal stage), (p = 0.975) and gender (p = 0.999). The regression coefficient for VDAC1 was 29.18 indicating a very poor prognosis for patients with high levels of VDAC1.The sample numbers were low for stages II and III with 68 samples being Stage II and only 16 samples being Stage III, this made it difficult to obtain statistically significant results. The correlation between VDAC1 expression and survival in stage II samples and Stage III samples was insignificant with p = 0.853 and p = 0.701 respectively. We had an inadequate number of events in both stages to produce an accurate Kaplan Meier plot with less than 10 incidences of death per category. The lack of higher stage samples led us to focus on the output from Stage I samples and from the combined data.

### VDAC1 overexpression correlates with a conserved 6-gene signature

**Figure 2:**
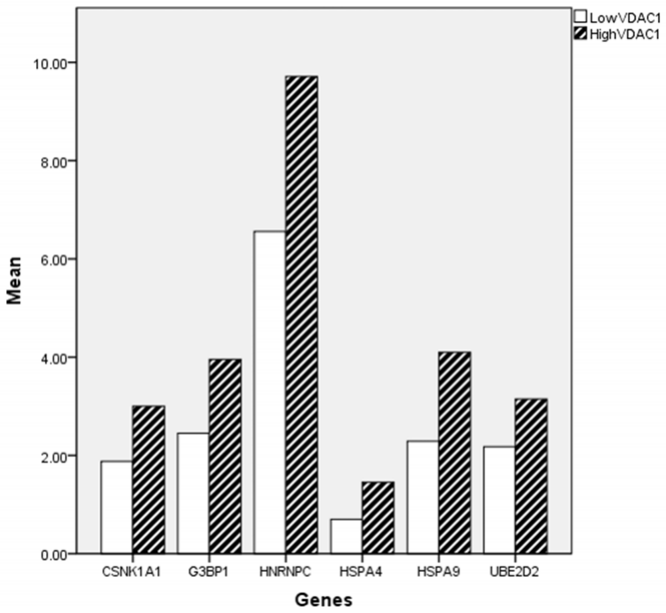
Change in gene expression of our six significantly differentially expressed genes linked to VDAC1 expression. This chart shows the variation in gene expression of our differentially expressed genes in the samples where VDAC1 expression is low and when it is high. All the genes that collectively made up our signature are upregulated and so increase when VDAC1 increases.

In order to gain an insight into the biology underlying the poor prognosis of VDAC1 overexpressing NSCLC in vivo, an analysis was conducted to identify which genes and gene networks are consistently associated with high VDAC1 expression. A subset of 6 genes was identified in VDAC1 overexpressing NSCLC datasets associated with significant differential expression in 50% or more of the datasets. These genes were CSNK1A1, G3BP1, HNRNPC, HSPA4, HSPA9 and UBE2D2; see Figure 2 where the difference in the average expression of these genes when VDAC1 is low and high is shown. As defined by the Gene Ontology Consortium [27] HNRNPC, HSPA4, HSPA9 and UBE2D2 are involved in protein ubiquitination pathways, and CSNK1A1 regulates EIF2 function. HSPA9 is also involved in anti-apoptosis pathways while G3BP1 is linked to Ras protein signal transduction and ATP dependent DNA and RNA helicase activity. CSNK1A1, HSPA4 and HSPA9 are linked to ATP binding while HNRNPC is involved in RNA and nucleotide binding. A summary of the GO terms for these genes can be found in Table 3. HNRNPC, HSPA9, G3BP1 and UBE2D2 were determined as being prognostic individually when a univariate analysis was carried out on their expression levels and survival time, the associated p values were 0.027, 0.002, 0.02 and 0.003 respectively.

**Table 3:** A table representing the gene ontology processes that define the genes in our meta-signature.

	Ubquitination/chaparone	RAS Signalling	P53 Regulation	Translation	RNA binding
**HNRNPC**					X
**UBE2D2**	X				
**HSPA4**	X				
**HSPA9**	X	X	X		
**CSNK1A1**				X	
**G3BP1**		X	X		

Any genes involved in the same processes are easily identifiable and overlap is evident. Ubiquitination is the most common known process shared by the significant genes; however RAS signalling and P53 regulation are also linked to 2 of the genes from the signature.

### Unsupervised analysis identifies VDAC1 as prognostic and associated with a 6-gene signature

We also analysed the data without filtering for VDAC1 expression to reaffirm VDAC1 would be significantly differentially expressed when expression levels of living and dead patients are compared. Using this unsupervised method and ranking gene expression according to overall survival time, VDAC1 was found to be downregulated in patients with longer survival. Similarly the 6 genes that are regulated alongside VDAC1 behaved in the same way, each of them were downregulated in samples from patients that survived for a long period of time.

### 6-gene Signature association with VDAC1 overexpression is independent of cancer type

To confirm the validity and potential biological relevance of the 6-gene signature covariant with VDAC1 we tested its discriminative power on an independent breast cancer dataset. A t-test was performed comparing high and low VDAC1 expressing samples. We found all 6 genes were upregulated when VDAC1 was highly expressed. Similarly, expression was low when VDAC1 expression was low and so our signature of genes covariant with VDAC1 in NSCLC data exhibited the same expression patterns in breast cancer data. We also carried out the same test using an independent myeloma cancer dataset, performing a t-test on the data which was split based on VDAC1 high and low expression. We again found all of the 6 genes in our meta-signature returned as being statistically significant at a p<0.001 level. Additional validation was found through the analysis of the NCI60 cell lines found in GSE2003. We again compared the samples based on VDAC1 expression and found the 6-gene signature was conserved in 8 of these cell lines, namely UACC62 and SK_MEL2 (melanoma), SF295 and SF539 (CNS), A498 and SNC12C (renal), H23 and HOP62 (NSCLC). Analysing the NCI60 data for low VDAC1 with low expression of the 6-gene signature identified 5 cell lines, CCRF-CEM, RPMI-8226 and K562B (leukaemia), IGROV1 (ovarian) and H522 (NSCLC).

### Validation of 6-gene signature using Principal Components Analysis (PCA)

PCA helps to explain the variance in data and is a common technique for dimensionality reduction in high dimensional data. We performed the PCA on the NSCLC dataset GSE3141 and found that although there were some outliers using the first principal component, which accounted for as much of the variability as possible, all 6 of the genes in our meta-signature were in the top 1% based on PC1 component loadings. HNRNPC and G3BP1 performed particularly well being placed fourth and fifth in the list respectively.

### Protein-Protein Interaction (PPI) Networks

Within the VDAC1 PPI network, using Yu et al's [28] categorisation, encoded proteins were classed as either hubs (nodes with high degree values constituting vulnerable areas of the network) and/or bottlenecks (those with high betweenness centrality scores corresponding to key intersecting nodes). VDAC1 and four of the genes, CSNK1A1, HSPA4, HNRNPC and UBE2D2 were considered as both hub-bottlenecks.

Clustering identified potential groups of genes operating in the same pathways, processes or molecular complexes. Five of the genes, G3BP1, UBE2D2, VDAC1, CSNK1A1 and HSPA4 belonged to four high-scoring clusters, the highest-ranked of these containing both UBE2D2 and G3BP1. Functional analysis revealed this cluster to be most significantly associated with the SH2 domain, ATP binding sites, protein and protein tyrosine kinase activity, autophosphorylation, DNA binding and transcription factor activity. The next most significant VDAC1-related cluster included HSPA4 which was enriched in nuclear lumen and nucleoplasm components and RNA-binding and mRNA splicing processes. VDAC1 itself was associated with a top-scoring cluster functionally enriched in regulation of programmed cell death, apoptosis, anti-apoptosis, nucleoplasm, intracellular organelle lumen, transcription factor binding and repressor activities. The cluster of which CSNK1A1 was a member was associated with significant functionality in coated pit, endomembrane system, endocytosis, endosome, binding and transport activities.

**Figure 3:**
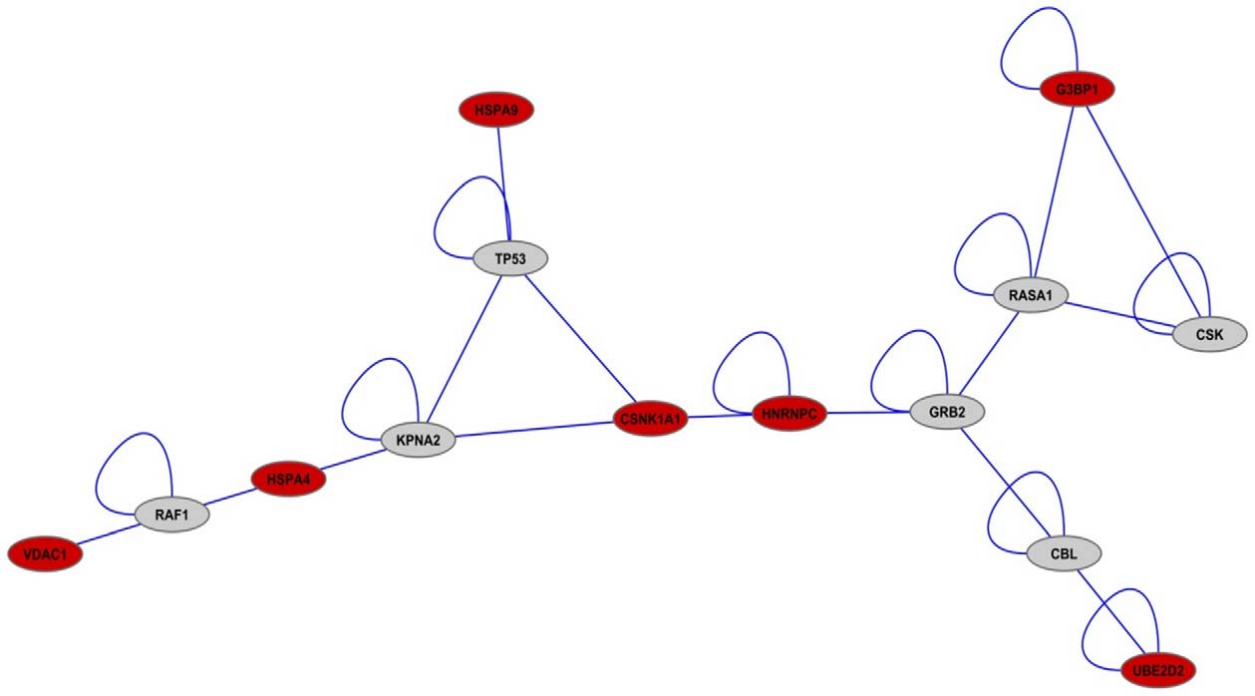
Shortest-path PPI analysis of VDAC1 target genes. These are shown with most-frequently occurring inter-connecting genes. Shortest path analysis revealed a sub-network of 14 genes, comprising VDAC1 and the 6 differentially expressed genes associated with VDAC1 expression. The key inter-connecting partners included TP53, RASA1, GRB2, CBL, CSK, RAF1 and KPNA2. Only HNRNPC and CSNK1A1 interacted directly.

Shortest path analysis revealed a sub-network of 14 genes, comprising the VDAC1 signature genes and key inter-connecting partners including TP53, RASA1, GRB2, CBL, CSK, RAF1 and KPNA2, see Figure 3. Only two of the genes HNRNPC and CSNK1A1 were direct interactors; HNRNPC's binding ability being modulated by CSNK1A1-mediated phosphorylation.

Functional analysis revealed enriched processes primarily associated with the VDAC1 target genes including ATP-binding and nucleotide binding. Both the VDAC1-target and internal shortest-path genes jointly participated in a number of significantly enriched processes and pathways. These included signalling processes (intracellular signalling cascade, RAS protein signal transduction, small GTPase mediated signal transduction), transport (nucleocytoplasmic transport, nuclear transport, intracellular protein transport) and anti-apoptosis.

The genes providing internal links within the shortest-path network were significantly enriched in signalling pathways, e.g. Neurotrophin, IGF-1, Insulin, TPO, PDGF, EGF, Integrin, T Cell Receptor, MAPK and ERKB. Further pathways identified included: signalling of hepatocyte growth factor receptor, transmembrane receptor protein tyrosine kinase signalling pathway, enzyme linked receptor protein signalling pathway, sprouty regulation of tyrosine kinase signals and IL-2 Receptor Beta Chain in T cell Activation.

The main combined activities of the VDAC1 genes (CSNK1A1, G3BP1, HSPA4, HSPA9 and UBE2D2) were focused on ATP and nucleotide binding. Specific individual enriched functionality also occurred, e.g. protein kinase activity and host-virus interaction (associated with genes CSNK1A1 and VDAC1, respectively).

Interestingly, although less prominent within the PPI network, the two non-hub-non-bottleneck genes, HSPA9 and G3BP1, were enriched in distinct sets of functions. In the case of G3BP1, signal transduction was highlighted (both small GTPase and RAS protein) in conjunction with the connecting genes, RAF1 and GRB2. HSPA9, in particular, was associated with two groups of functions: protein targeting and transport (intracellular protein, nuclear and nucleocytoplasmic) in conjunction with TP53 and KPNA2; and negative regulation of cell death, apoptosis and programmed cell death (together with TP53 and RASA1).

## Discussion

VDAC1 overexpression predicts shorter time to recurrence and overall survival for NSCLC as evidenced from pooled gene expression analysis. It is an independent prognostic factor as evidenced from Cox regression analysis and predicts survival in stage 1 disease, where subgroup analysis of randomized controlled clinical trials of adjuvant chemotherapy have failed to show clinical benefit [24], [25], [26]. A combined gene expression data analysis approach has been employed to investigate the impact of VDAC1 expression on survival, which was statistically significant in all individual datasets examined.

Several recent studies analysing microarray data for prognostic markers in NSCLC have produced inconsistent results [29]. The distinct lack of overlap associated between these signatures reflects instability and is attributed to small sample sizes with less than 200 samples used per study. Analysis of multiple datasets, for example combined analysis of microarray gene expression datasets addressing similar biological questions conducted at an interpretational level by meta-analysis, can enable more accurate results. Many studies propose methods for meta-analysis of microarray data with the aim of identifying significantly differentially expressed genes across studies using statistical techniques that avoid the direct comparison of gene expression values [30]. The evaluation of multiple datasets as employed in this study has been shown to yield more reliable and valid results, because they are based on large sample numbers and the individual bias caused by each study is weakened [30].

It is not known why VDAC1 correlates with poor survival outcomes. To understand the most relevant genetic features of VDAC1 overexpressing NSCLC, we conducted a gene expression meta-analysis [31], [32] to identify a subset of genes (signature) that were significantly enriched. We employed stringent statistical criteria, combined with a large sample size to support the identification of these VDAC1-covariant genes. VDAC1 and 6 gene signature was then validated across breast, myeloma and NCI-60 datasets [33], suggesting enrichment of genes which were independent of the type of cancer.

Interestingly, of the 6 genes identified as being conserved and significantly differentially regulated in the high VDAC1 expressing group, most were functionally linked to the regulation of protein turnover. These genes included heat shock 70kDa protein 4 (HSPA4), ubiquitin-conjugating enzyme E2D 2 (UBE2D2), and heat shock 70kDa protein 9 (mortalin/HSPA9), encoding a glucose regulated 75 kilodalton protein previously reported as correlating with poor survival in colorectal cancer [34]. HSPA9 also binds and inactivates wild type p53 [35], and regulates the RAS RAF MEK pathway[36]. Similarly, p53 and RAS are targets of GTPase activating protein (SH3 domain) binding protein 1 (G3BP1) [37], and shown to predict shorter survival in oesophageal cancer [38]. Casein kinase 1, alpha 1 (CSNK1A1) regulates protein turnover via initiation of translation via EIF2 and participates in wnt signalling; a homologue CSKNK2A1 has been previously identified as an independent predictor of survival in squamous cell lung cancer [39]. Like G3BP1, heterogeneous nuclear ribonucleoprotein C (C1/C2) or HNRNPC is involved in RNA binding and transcription [40].

The PPI networks identified that the main combined activities of the VDAC1 genes (CSNK1A1, G3BP1, HSPA4, HSPA9 and UBE2D2) were focussed on ATP and nucleotide binding. Specific individual enriched functionality also occurred, e.g. protein kinase activity and host-virus interaction (associated with genes CSNK1A1 and VDAC1, respectively). Interestingly, although less prominent within the PPI network, the two non-hub-non-bottleneck genes, HSPA9 and G3BP1, were enriched in distinct sets of functions. In the case of G3BP1, signal transduction was highlighted (both small GTPase and RAS protein) in conjunction with the connecting genes, RAF1 and GRB2. HSPA9, in particular, was associated with two groups of functions: protein targeting and transport (intracellular protein, nuclear and nucleocytoplasmic) in conjunction with TP53 and KPNA2; and negative regulation apoptosis (together with TP53 and RASA1).

### Conclusions

In summary, based on the poor prognosis associated with overexpression of VDAC1 and the associated 6-gene signature, methods to target this molecular subclass of NSCLC may be effective in improve survival outcomes after surgery. We have identified cell lines which have upregulation of the 6-gene signature found in VDAC1 overexpressing NSCLC. Ongoing work aims to target this signature to determine the effects on cell viability utilizing the connectivity map. We propose that regulating the expression of VDAC1 and/or genes with the 6-gene signature, could provide a novel therapeutic strategy for targeting poor risk patients with NSCLC.

## Materials and Methods

### Gene expression datasets

To conduct gene expression meta-analysis, a library of 8 NSCLC gene expression datasets was curated from NCBI Gene Expression Omnibus (GEO) [41]: GSE8894, GSE3141 (array type: HG-U133 Plus2.0), GSE6253, GSE4573 (array type: HG-U133A), GSE6253 (array type: HG-U133B), GSE6253 (array type: HG-U95Av2), GSE4716 (array type: GeneFilter Human Microarray Release II) and GSE5123 (array type: PC Human Operon v 2 21k). They had 602 samples in total and contained gene expression information on primarily resected tumour samples that had received no previous treatment. They were pre-processed using R [42] and Bioconductor [43]. Normalization was carried out using the RMA algorithm [44] as implemented in Bioconductor. Data was base-two log-transformed where applicable. Along with gene expression measurements, there were also recordings of patient information, including age, sex, histology and overall survival time, and in some cases stage, recurrence free survival time, tumour stage (pT) and nodal stage (pN) were also recorded. The genes that were found to be significantly differentially expressed in the NSCLC datasets were then validated in independent breast cancer datasets obtained from GEO: GSE6434 containing HG-U95Av2 array data from 24 patients who had received chemotherapy and GSE2034 with HG-U133A array data from 286 samples. We also tested the meta-signature in a myeloma dataset (GSE2658) with HG-U133 Plus2.0 array data from 559 patients who had received chemotherapy. Further validation was found through the analysis of the NCI60 cell line panel (GSE2003) were we checked gene expression matched what we found in our subset of significant genes. Additionally we checked our results by carrying out principal components analysis on the data in GSE3141.

### Univariate and multivariate survival analysis

The distribution of VDAC1 expression was subdivided into tertiles for each data set, corresponding to low, medium and high. Kaplan-Meier curves were plotted using Prism (GraphPad Software, San Diego California USA, www.graphpad.com) based on survival data, comparing the highest versus lowest tertile for VDAC1, initially these plots were produced for all data and then only for stage I. Hazard ratios comparing low and high VDAC1 expressing samples were calculated. We carried out correlation analysis between VDAC1 expression and survival time using Spearman's Rank correlation coefficient. We also performed univariate and multivariate analysis using PASW (SPSS: An IBM company), recurrence-free and overall survival were used as clinical endpoints to assess prognostic significance of VDAC1; the logrank ratio was considered to be significant if p<0.05. Cox multivariate analysis was used to determine whether VDAC1 is an independent prognostic variable in relation to 7 clinical variables: age, gender, stage, histology, tumour size (pT), nodal stage (pN) and survival.

### Supervised microarray meta-analysis

We adopted the method termed meta-analysis of microarrays to determine what genes were regulated alongside VDAC1 [45]. Each dataset was subdivided according to VDAC1 expression into tertiles; we compared the top and bottom VDAC1 tertiles samples, using TMeV [46]. Each gene was assessed for differential expression using a two class t-test for each dataset with class 1 being low VDAC1 expression and class 2, high VDAC1 expression. Bonferroni's correction was also used and this analysis was carried out on all the 8 datasets. Bonferroni's correction helps minimise the likelihood of generating false positives. The number of probes is vast with some datasets containing more than 54,000, so to reduce our significant genes to a manageable list per dataset we used a stringent cut off criteria where a gene was labelled as significantly differentially expressed if the t-test returned a p value of less than 0.001. The same hypotheses were tested in each NSCLC dataset independently e.g. genes differentially expressed based on VDAC1 expression. To enable multiple hypothesis testing, we used a method that compares statistical measures and Q values (estimated false discovery rates) calculated independently from each dataset [32], [47], where
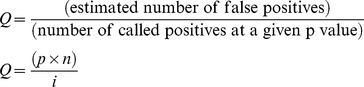



Where p is the probability, n is the total number of genes and i is the sorted rank of the p value. The strict p value cut-off we applied was carried forward when Q values were calculated. After ranking the significantly differentially expressed genes based on their Q value a direction and significance threshold of Q<0.10 was set to define differential expression signatures from the already computed differential expression analysis. Having obtained an ordered list of differentially expressed genes for each dataset genes were then filtered based on the number of datasets in which they were differentially expressed.. The genes that were defined as being significantly differentially expressed were then validated in independent breast cancer datasets (GSE6434 and GSE2034) obtained from GEO with 24 and 286 samples respectively. We also tested the meta-signature in a myeloma dataset (GSE2658) with 559 samples. T-tests were performed on this additional data; we compared high and low VDAC1 expressing samples to determine if the genes from our signature were identified once again. The same procedure was used on the NCI60 cell lines to identify cell lines in which expression levels matched our signature.

### Protein protein interaction network analysis

Using VDAC1 and the six associated SDE genes as seeds together with their interactions found in the Human Protein Reference Database (HPRD) [48], a parent PPI network (including recursive interactions) was created using an in-house Java-based program. Using these seed proteins as the initial depth (level 0), subsequent depths were obtained using snowball sampling. The resulting parent PPI network (9,266 gene nodes, 38,800) was then viewed, manipulated and analyzed in Cytoscape [49]. Using the Network Analyzer plug-in [50] gene nodes were then identified as either hubs or bottlenecks using the classification of Yu et al [28]; the top 20% of nodes by degree value were identified as hubs, the remainder as non-hubs. The same ranking method was applied to the betweenness centrality metric to identify bottleneck nodes. Each node in the PPI network was thus classified in one of four categories: non-hub-non-bottlenecks (NH/NB), hub-non-bottlenecks (H/NB), non-hub-bottlenecks (NH/B), and hub-bottlenecks (H/B). Multiple shortest paths between the seven genes were identified using the PeSca tool. Key shortest paths were identified by considering direct connections between the target genes, paths in which the target genes themselves acted as inter-connectors, and the most frequently occurring genes providing internal linkages. Modular functional enrichment analysis of the gene clusters and shortest path network was carried out using the online tool DAVID [51].
